# Whispers of Loss, Echoes of Strength: The Experience of Bedouin Arab Grandparents Who Lost a Grandchild in War

**DOI:** 10.1093/geroni/igaf122.256

**Published:** 2025-12-31

**Authors:** Reem Nashef-Hamuda, Hadass Goldblatt

**Affiliations:** University of Haifa, Tel Aviv University, Haifa, HaZafon, Israel; University of Haifa, Haifa, HaZafon, Israel

## Abstract

There are 47 unrecognized Bedouin villages located in the ‘open areas’ of the Negev in southern Israel that lack protection from the Iron Dome missile defense system. Having no safe shelters or permanent houses, these communities are more prone to direct injury from rocket fire, resulting in a more significant number of casualties. During the war, 19 civilians from the Negev Bedouin Arab population were killed, including six children (Arava Institute, 2023). The purpose of this study is to explore in-depth the bereavement experience of Bedouin grandparents following the death of a grandchild during the war. We examined this phenomenon from an intersectionality perspective, emphasizing the overlap between minority status, advanced age, and the experience of bereavement. This study employed a qualitative narrative approach. Data collection consisted of in-depth, semi-structured interviews with 10 Bedouin grandparents who had lost grandchildren during the war. Interviews were conducted in Arabic to ensure a culturally sensitive exploration of the grandparents’ experiences. Data were analyzed using thematic analysis. Two themes will be presented: (1) “When it’s war and sudden, it’s harsh:” The experience of traumatic loss and accompanying emotions following bereavement; (2) “God gives you the strength to endure the suffering:” Faith in God as a source of meaning and comfort. These themes illustrate the dual reality of grief and resilience in a community shaped by conflict. This study provides a deeper understanding of bereavement processes among minority groups in conflict settings, offering insights into culturally sensitive coping mechanisms.

